# MYCN drives glutaminolysis in neuroblastoma and confers sensitivity to an ROS augmenting agent

**DOI:** 10.1038/s41419-018-0295-5

**Published:** 2018-02-14

**Authors:** Tingting Wang, Lingling Liu, Xuyong Chen, Yuqing Shen, Gaojian Lian, Nilay Shah, Andrew M Davidoff, Jun Yang, Ruoning Wang

**Affiliations:** 1 Center for Childhood Cancer and Blood Diseases, Hematology/Oncology and BMT, The Research Institute at Nationwide Children’s Hospital, The Ohio State University, Columbus, OH USA; 2 Department of Surgery, St. Jude Children’s Research Hospital, Memphis, TN 38105 USA

## Abstract

Heightened aerobic glycolysis and glutaminolysis are characteristic metabolic phenotypes in cancer cells. Neuroblastoma (NBL), a devastating pediatric cancer, is featured by frequent genomic amplification of MYCN, a member of the Myc oncogene family that is primarily expressed in the early stage of embryonic development and required for neural crest development. Here we report that an enriched glutaminolysis gene signature is associated with MYCN amplification in children with NBL. The partial knockdown of MYCN suppresses glutaminolysis in NBL cells. Conversely, forced overexpression of MYCN in neural crest progenitor cells enhances glutaminolysis. Importantly, glutaminolysis induces oxidative stress by producing reactive oxygen species (ROS), rendering NBL cells sensitive to ROS augmentation. Through a small-scale metabolic-modulator screening, we have found that dimethyl fumarate (DMF), a Food and Drug Administration-approved drug for multiple sclerosis, suppresses NBL cell proliferation in vitro and tumor growth in vivo. DMF suppresses NBL cell proliferation through inducing ROS and subsequently suppressing MYCN expression, which is rescued by an ROS scavenger. Our findings suggest that the metabolic modulation and ROS augmentation could be used as novel strategies in treating NBL and other MYC-driven cancers.

## Introduction

Heightened aerobic glycolysis (i.e., the “Warburg effect”) and glutaminolysis are characteristic hallmarks of cancer cells^1–5^. Both processes are tightly controlled to fulfill cell growth-associated and proliferation-associated bioenergetics, biosynthetic, and redox demands. While tissue microenvironments play a role in homeostatic regulation of cell metabolism, the metabolic rewiring of cancer cells is largely driven by a hierarchical oncogenic cascade involved in Akt/mTOR, mitogen-activated protein kinase signaling, and a hypoxia-inducible factor 1 (HIF1)-dependent and Myc-dependent metabolic transcriptome^4,6^. By analogy to the concept of oncogene addiction^7^, we envision that a persistent metabolic rewiring renders cancer cells highly dependent on certain metabolic pathways in a way that other cells are not (metabolic addiction), hence modulation of this process holds the promise of novel metabolic interventions (metabolic vulnerability).

Neuroblastoma (NBL) is an embryonal malignancy of early childhood, arising from sympathoadrenal precursors that have evaded terminal differentiation and proliferated uncontrollably. Approximately half of the patients with NBL are considered “high risk,” as defined by clinical, radiographic, and biological criteria. These patients have a high rate of treatment failure, most commonly due to disease progression early in treatment or relapse at the end of multimodal therapy. These failures make NBL the deadliest extracranial pediatric solid tumor, accounting for 15% of childhood cancer deaths^8,9^. Children with high-risk NBL are treated with aggressive multimodal therapy. Nevertheless, <50% of patients with high-risk NBL will survive long term with current therapies, and survivors are at risk for serious treatment-related late toxicities. Therefore, novel treatments must be developed to enhance therapy efficacy with minimal toxicity, prevent disease recurrence, and maintain durable cures.

While several genetic abnormalities (ALK, PHOX2B, Let-7, ATRX, PTPN11, etc.) are known to contribute to the pathogenesis of subsets of NBL, genomic amplification of the Myc oncogene family member, MYCN, occurs in about 50% of high-risk NBL cases and is the most prevalent genetic abnormality identified in NBL^10^. MYCN is a potent oncogenic driver and the single worst prognostic biomarker in NBL, with MYCN amplification indicating <30% chance of survival^11^. It has been suggested that MYCN regulates the transcription of some metabolic enzymes and transporters involved in MYCN-amplified NBL cell lines^12,13^. Also, activating transcription factor 4 (ATF4) and HIF1 are involved in regulating the transcription of metabolic genes in glutamine and glucose metabolic pathways, respectively^12,14,15^. The concept of metabolic reprogramming and its role in cell fate determination is well established in metabolic diseases, and, more recently, it has been applied to many adult cancers^3,16,17^. However, the impact of metabolic reprogramming of cancer cells by oncogenes is not entirely clear. How to harness the impact of metabolic reprogramming to develop novel therapies is also very important for cancer treatment. A better understanding of how genetic alterations (MYCN amplification) impact NBL metabolic reprogramming will enable us to identify key oncogenic events and metabolic characters, and to devise effective therapies.

Here, we report a role of MYCN in regulating NBL metabolic reprogramming and reactive oxygen species (ROS) induction. The short hairpin RNA (shRNA)-mediated partial knockdown of MYCN suppresses the expression of metabolic genes and the activity of glutaminolysis in NBL cell lines. Heightened glutaminolysis in NBL cells by MYCN provides bioenergetic support and induces ROS as a by-product in mitochondria, conferring metabolic vulnerability of NBL cells to ROS-producing agent as cancer cells are more sensitive, than normal cells, to agents that cause further accumulation of ROS. We identified dimethyl fumarate (DMF), a Food and Drug Administration (FDA)-approved drug for inflammation and autoimmunity, as a novel therapeutic agent that suppresses NBL cell growth through inducing ROS and subsequently suppressing MYCN expression. Our studies suggest that metabolic modulation of glutaminolysis and ROS augmentation may represent effective strategies in treating NBL and other MYC-driven cancers.

## Results

### MYCN is required for driving glutaminolysis in MYCN-amplified NBL

**Fig. 1:**
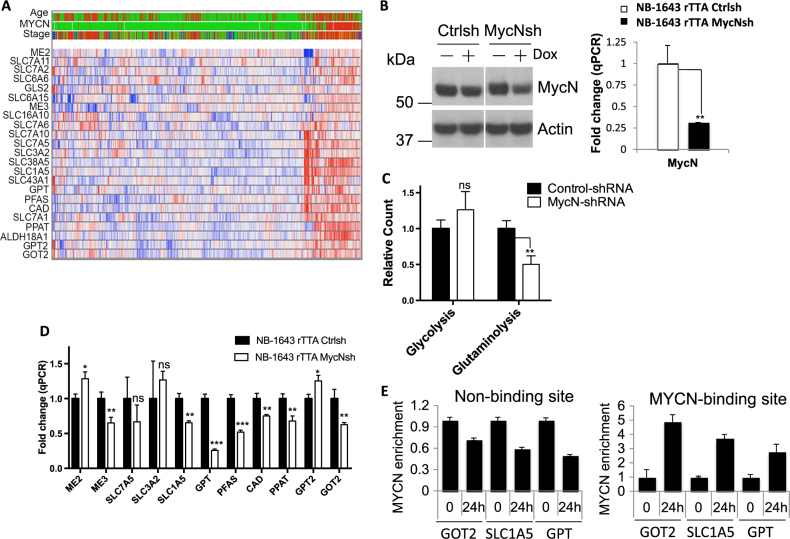
MYCN is required for driving glutaminolysis in neuroblastoma. **a** Genes in glutaminolysis pathway were upregulated in MYCN-amplified tumors (93 cases) compared with non-MYCN-amplified tumors (550 cases) (ANOVA, *p* < 0.01). These data were derived from Kocak dataset (GSE45547). Age (red > 18 months; green < 18 months); MYCN (red = amplification; green = non-amplification); stage (red = stage 4; blue = stage 4S; brown = stage 3; dark green = stage 2, green = stage 1). **b** MYCN protein and mRNA expression levels were determined by immunoblot (left panel) and qPCR (right panel), respectively. NB-1643 control-shRNA-inducible and MYCN-shRNA-inducible cells were collected after 3 days in the absence or presence of 1 μg/ml doxycycline for MYCN level analysis. mRNA levels in cells expressing control-shRNA were set to 1. Error bars represent standard deviation from the mean of triplicate samples. Data are representative of two independent experiments. **c** Glycolysis and glutaminolysis as determined by the generation of ^3^H_2_O from [5-^3^H]glucose and the generation of ^14^CO_2_ from [U-^14^C]glutamine, respectively. NB-1643 control-shRNA and MYCN-shRNA cells (collected after incubation with 1 μg/ml doxycycline for the expression of specific RNA in 2% FBS media for 3 days, followed by overnight 10% FBS media recovery) were used for measuring the metabolic flux. Error bars represent standard deviation from the mean of triplicate samples. Data are representative of two independent experiments. **d** mRNA levels of genes essential for glutaminolysis in NB-1643 cells were determined by qPCR. NB-1643 control-shRNA-inducible and MYCN-shRNA-inducible cell lines were treated with 1 μg/ml doxycycline for 3 days before harvesting the samples for RNA extraction followed by qPCR analyses of metabolic genes in the glutaminolytic pathway. mRNA levels in NB-1643 control-shRNA cells were set to 1. Error bars represent standard deviation from the mean of triplicate samples. Data are representative of two independent experiments. **e** Chromatin immunoprecipitation assessment of the binding of MYCN on indicated genes after 24-h MYCN induction with 500 ng/ml doxycycline in MycN3 cells. MYCN was immunoprecipitated from cross-linked DNA, and then DNA was extracted for PCR with primers that amplify predicted binding site and non-binding site for each gene

Although it has been well recognized that the proto-oncogene, MYC, is responsible for orchestrating a transcriptional program driving metabolic reprogramming in many adult tumors, the role of MYC, specifically its family member MYCN, during metabolic reprogramming in pediatric cancers including NBL is not fully understood. We, therefore, sought to determine whether MYCN is involved in regulating metabolic programs in MYCN-overexpressed NBL. By cross-referencing the published microarray and genetic data, we found that an enriched glutaminolysis gene signature is associated with MYCN amplification in children with NBL (Fig. 1a). Next, we sought to assess whether the genetic modulation of MYCN impacts NBL cell metabolism. The transient knockdown of MYCN by small interfering RNA (siRNA) did not allow us to obtain sufficient amount of cells required for metabolic assays. Therefore, we established NB-1643 (a NBL cell line with MYCN overexpression) stably expressing a doxycycline (Dox)-inducible MYCN-shRNA, which not only allows us to acutely knockdown endogenous MYCN level following Dox treatment, but also allows us to obtain sufficient amount of cells to examine metabolic activity following MYCN knockdown. Immunoblot (IB) and quantitative PCR (qPCR) analysis confirmed the modest downregulation of MYCN (Fig. 1b). Then, we utilized radiochemical-based approaches to assess the metabolic activities in control and MYCN-shRNA knockdown groups. The results showed that even modest downregulation of MYCN led to a 50% reduction in glutaminolysis rate compared to the control group, as indicated by ^14^CO_2_ release from [U-^14^C]glutamine (Fig. 1c). Meanwhile, the rate of glycolysis, measured by the detritiation of [5-^3^H]glucose, did not differ significantly between the two groups, suggesting that glutaminolysis is more sensitive to the loss of MYCN than glycolysis. Consistent with a reduced glutaminolysis activity, the expression of metabolic genes involved in glutaminolysis is dampened to different extents following acute induction of MYCN-shRNA knockdown (Fig. 1d and Fig. S1A). These results were supported by a different shRNA knockdown of MYCN in NB-1643 cells (Fig. S1C, S1D), which resulted in suppression of glutaminolysis but not glycolysis (Fig. S1C), despite that the second shRNA had a lower knockdown efficiency. To further cross-validate that MYCN is responsible for regulating glutaminolysis, we took additional step-wise approaches. We first transfected NB-EBC1 (MYCN-overexpressed NBL cell line) with MYCN siRNA, and confirmed that siRNA-mediated knockdown of MYCN in NB-EBC1 also resulted in the downregulation of metabolic genes involved in glutaminolysis, albeit with some discrepancies on genes including *ME2*, *SLC7A5*, and *SLC3A2*, compared to the result in NB-1643 (Fig. S1B and Fig. 1d). We reasoned that the difference in cell lines (NB-1643 vs. NB-EBC1) and knockdown methods (shRNA vs. siRNA) likely contributed to these discrepancies. Next, we used a gain-of-function approach to further strengthen the conclusion that MYCN plays a role in regulating glutaminolysis. We first employed a recently established cellular model based on the genetic manipulation and transformation of the JoMa cells, a murine multipotent neural crest progenitor cell line^18,19^. JoMa cells can be kept in an undifferentiated state, differentiated into a variety of cell lineages, or transformed into NBL cells by MYCN overexpression in vitro and developed to NBL after transplantation in vivo^18,19^. This model allowed us to analyze the molecular and metabolic alterations in NBL progenitor cells. As such, we generated a lentiviral construct to overexpress MYCN to mimic MYCN amplification in NBL. Our results showed that the overexpression of MYCN in JoMa cells promotes glutaminolysis, but not glycolysis in these cells (Fig. S1E). Then, we used a Dox-inducible MYCN cell line (MycN3)^20^ and further showed that acute induction of MYCN significantly elevated glutaminolysis but not glycolysis in NBL cells (Fig. S1F). Finally, we sought to examine whether MYCN directly controls the transcription of the metabolic genes involved in glutaminolysis. Again, we employed the MycN3 cell line that enabled us to acutely induce the expression of MYCN, thereby assessing the binding of MYCN on the predicated binding or non-binding site (intrinsic negative control) in each candidate gene by chromatin immunoprecipitation and polymerase chain reaction (ChIP-PCR). After 24 h induction, MYCN binding was greatly enriched in the promoter regions but not negative control sites (predicated non-binding sites) of the genes including *GOT2*, *SLC1A5*, and *GPT* that we randomly chose to examine (Fig. 1e). Together, these results suggest that MYCN plays a critical role in the regulation of glutaminolysis in NBL.

### Glutaminolysis increases the production of ROS and renders NBL cells sensitive to oxidative stress

**Fig. 2:**
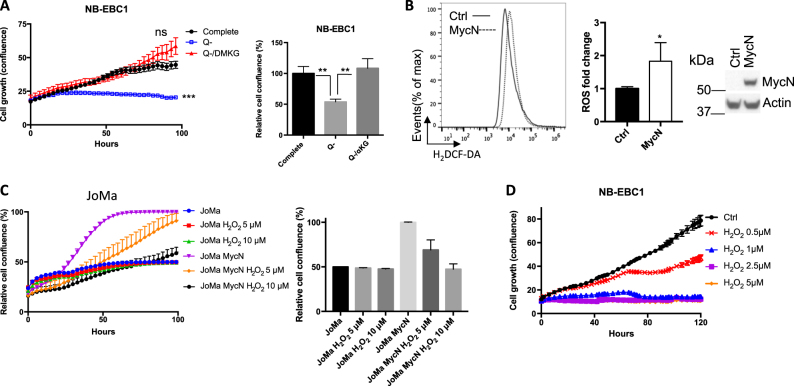
Glutaminolysis elevates intracellular ROS and confers senstivity of NBL cells to ROS augmentation. **a** NB-EBC1 cell proliferation curves under indicated culture conditions were determined by live-cell imaging analysis (IncuCyte ZOOM™). Cell confluences of complete media at 96 h were set to 100%. Error bars represent standard deviation from mean of quadruplicate samples. **b** JoMa cells were transduced with lentiviral vector carrying mouse MYCN gene or blank control plasmid and cultured for 3 days. The ROS level (left) was determined by flow cytometry in cells followed with 30 min incubation with 5 μM CM-H_2_DCFDA at 37 °C. The ROS level in control JoMa cells were set to 1 in the bar graph. Error bars represent standard deviation from the mean of triplicate samples. MCYN protein levels (right) were determined by immunoblot. Data are representative of two independent experiments. **c** JoMa cells were transduced with lentiviral vector carrying mouse MYCN gene or blank control plasmid for 3 days and the growth curve of cells treated with indicated doses of H_2_O_2_ was determined by live-cell imaging analysis (IncuCyte ZOOM™). **d** NB-EBC1 cell growth upon treatment of H_2_O_2_ in different concentrations was determined by live-cell imaging analysis (IncuCyte ZOOM™)

Previous studies demonstrated that glutamine is an essential metabolic substrate driving cancer cell growth through fueling mitochondrial oxidation and providing carbon and nitrogen for the biosynthesis of other macromolecules^2^. Consistent with these studies, our results showed that the removal of glutamine from culture medium significantly dampened the growth of MYCN-overexpressed NBL cells. However, the supplement of dimethyl α-ketoglutarate (DMKG, cell-permeable analog of α-KG), a downstream metabolite of glutamine, restored cell growth in the absence of glutamine (Fig. 2a and Fig. S2A). These results suggest that glutaminolysis supports NBL cell growth by providing α-KG, an anaplerotic substrate of the tricarboxylic acid (TCA) cycle, fueling oxidative phosphorylation in mitochondria. It is known that enhanced oxidative phosphorylation through mitochondrial electron transport chain (ETC) produces ROS as a by-product, which is required for promoting cell proliferation and transformation but also leaves cells more vulnerable to further oxidative stress^21,22^. Therefore, we further investigated whether MYCN-dependent glutaminolysis impacted ROS production and cellular response to oxidative stress. Our results showed that overexpression of MYCN in JoMa cells increased the fluorescent intensity of H_2_DCFDA (DCF) (Fig. 2b), indicating elevated levels of intracellular hydrogen peroxide (H_2_O_2_) that is the end product following ROS disputation. As illustrated in Fig. 2c, the addition of H_2_O_2_ suppressed the proliferation of MYCN-transformed JoMa cells but not control JoMa cells. Consistent with this, H_2_O_2_ treatment significantly suppressed the proliferation of MYCN-overexpressing NBL cell lines (Fig. 2d and Fig. S2B). We further discovered that the removal of glutamine from culture media (glutamine starvation) or the addition of glutaminolysis inhibitor 6-diazo-5-oxo-l-norleucin (DON) significantly reduced ROS levels in MYCN-overexpressing JoMa cells (Fig. S2C). Together, our data suggest that MYCN-driven glutaminolysis increases ROS generation and sensitizes NBL cells to oxidative stress.

### Identification of DMF that suppresses NBL cell proliferation

**Fig. 3:**
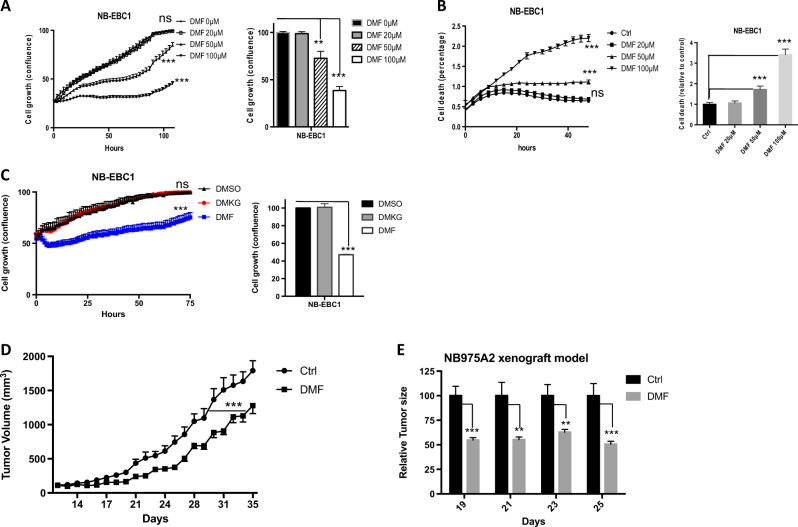
Dimethyl fumarate suppresses NBL cell growth. **a**–**c** NB-EBC1 cell growth (**a** and **c**) and cell death (**b**) upon indicated treatment were determined by live-cell imaging analysis (IncuCyte ZOOM™) (right panel). The cell confluences or death percentage of control group were set to 100% or 1 in the bar graph (left panel), respectively. Error bars represent standard deviation from the mean of quadruplicate samples. **d**, **e** Tumor xenograft was established by using NB975A2 cells and mice were treated daily with 300 mg/kg DMF or vehicle controls. The tumor volumes of control group and DMF-treated group were monitored daily. The tumor volumes of vehicle control group were set to 100%. Error bars represent SEM from the mean of 10 tumors. Data are representative of three independent experiments

Next, we selected a list of compounds with activities against a range of cell catabolic pathways and examined their impact on NBL cell growth (Supplemental Table 1). While some compounds suppressed the growth of MYCN-overexpressed NBL cell lines (Fig. S3A), we chose to follow-up with DMF, which exhibited strong inhibition of NBL cells growth and was also suggested to induce ROS through binding and depleting intracellular glutathione (GSH)^23,24^. Our gene enrichment analysis revealed an enriched oxidative response gene signature in DMF-treated NBL cells (Fig. S3B), confirming that DMF treatment induces oxidative stress. DMF is the key active ingredient of BG-12/TECFIDERA and FUMADERM; both have been approved for treatment against the autoimmune diseases multiple sclerosis (MS) and psoriasis^23,24^. As previous studies showed that DMF exhibited anti-inflammatory activities against immune cells with doses ranging from 20 to 100 μM^25,26^, we applied a similar dose range to NBL cells and observed a dose-dependent suppression of cell growth and induction of cell death (Fig. 3a, b and Fig. S3C). Given that DMF is a cell-permeable form of fumarate, a TCA cycle metabolite, we then investigated whether other TCA cycle metabolites displayed similar anti-tumor activity as DMF does. DMKG, a cell-permeable form of α-ketoglutarate, failed to suppress NBL cell growth (Fig. 3c and Fig. S3D), suggesting the anti-tumor property is unique in DMF but not in other TCA cycle metabolites. As fumarase (FH) converts fumarate to malate during the TCA cycle, we hypothesized that the knockdown of FH increases fumarate and consequentially suppresses NBL cell growth. To test this hypothesis, we transiently transfected NBL cells with siRNAs targeting FH. As shown in Fig. S3E, FH siRNAs partially reduced the expression of FH in NBL cells and delayed NBL cell growth. Next, we sought to determine whether DMF had any anti-tumor activities in vivo. Given the known autoimmune-suppressive effect of DMF, we reasoned that testing DMF in an immune-deficient host might potentially exaggerate its anti-tumor effect. Therefore, we assessed the tumor-suppressive efficacy of DMF in a syngeneic NBL xenograft mouse model, which is based on a mouse NBL cell line derived from TH-MYCN transgenic mice, allowing us to establish NBL xenograft in immunocompetent hosts^27^. Our data showed that DMF treatment significantly delayed NBL tumor growth in this model (Fig. 3d, e). In addition to its known immune-suppressive activities, our data have shown that DMF exhibited the anti-tumor activity in MYCN-overexpressing NBL cell lines and in mouse NBL xenografts.

### DMF suppresses the expression of MYCN through augmenting ROS

**Fig. 4:**
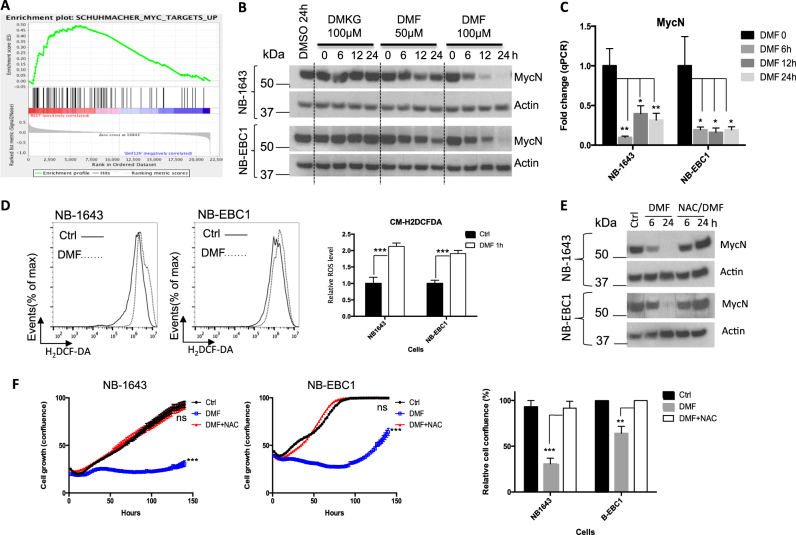
DMF augments oxidative stress and suppresses MYCN expression. **a** NB-1643 cells were incubated with 100 μM DMF for 12 h and harvested for RNA extraction for microarray analysis followed with gene set enrichment analysis. **b** The expression of indicated proteins in NB-1643 and NB-EBC1 cells was determined by immunoblot. Data are representative of two independent experiments. **c** MYCN mRNA levels in NB-1643 and NB-EBC1 cells upon indicated treatments were determined by qPCR. MYCN mRNA levels in control cells were set to 1. Error bars represent standard deviation from the mean of quadruplicate samples. **d** NB-1643 and NB-EBC1 cells were incubated with 100 μM DMF for 1 h. The ROS level was determined by flow cytometry in cells followed by 30 min incubation with 5 μM CM-H_2_DCFDA at 37 °C. ROS levels in control cells were set to 1. Error bars represent standard deviation from the mean of triplicate samples. **e** NB-1643 and NB-EBC1 cells were treated with 100 μM DMF in the absence and presence of 5 mM ROS scavenger NAC and collected at designated time points. The expression of indicated proteins was determined by immunoblot. **f** NB-1643 and NB-EBC1 cells were treated with 100 μM DMF in the absence and presence of 5 mM ROS scavenger NAC. Cell proliferation was determined by live-cell imaging analysis (IncuCyte ZOOM™). Cell confluences of control groups at the end of day 6 were set to 100% in the bar graph. Error bars represent standard deviation from the mean of quadruplicate samples

To gain more mechanistic insights on the effects of DMF on NBL cells, we analyzed our gene expression data and found that a number of Myc target gene signatures were significantly suppressed following DMF treatment (Fig. 4a). Our results showed that the transient knockdown of MYCN suppressed NBL proliferation, confirming the role of MYCN as a key oncogenic driver in these cell lines (Fig. S4A, S4B). Next, we examined the level of MYCN in NBL cell lines following DMF treatment. As Fig. 4b, c showed, DMF suppresses the expression of MYCN at both the mRNA and the protein levels. To further explore the molecular mechanisms behind the suppression of MYCN expression and NBL growth upon DMF treatment, we assessed the levels of ROS following DMF treatment. Consistent with the induction of oxidative response genes (Fig. S3B), DMF treatment increased the intracellular ROS levels in NBL cells (Fig. 4d). We then treated NBL cells with H_2_O_2_ to mimic the accumulation of intracellular ROS. Consistent with the effect on cell growth (Fig. 2d and Fig. S2B), H_2_O_2_ treatment reduced the levels of MYCN in a dose-dependent manner (Fig. S4C), indicating that increased ROS suppressed MYCN expression. To determine whether ROS mediates the effects of DMF on NBL cells, we concurrently treated NBL cells with DMF and *N*-acetyl-l-cysteine (NAC), an ROS scavenger. As Fig. 4e, f showed, NAC restored the level of MYCN and the proliferation of NBL cells following DMF treatment. In addition, GSH reduced ethyl ester (GSH-MEE), a membrane-permeable form of antioxidant, also protected NBL cells against the DMF-mediated anti-proliferation effect (Fig. S4D). Finally, we sought to examine whether genetic knockdown of FH suppresses NBL cell proliferation through ROS induction. The addition of NAC had no impact on cell growth in control group; however, it partially restored cell growth in the FH siRNA group (Fig. S4E). Collectively, our results suggest that DMF suppresses MYCN expression and cell growth through augmenting the level of ROS.

### DMF induces a compensatory NRF2-mediated response

**Fig. 5:**
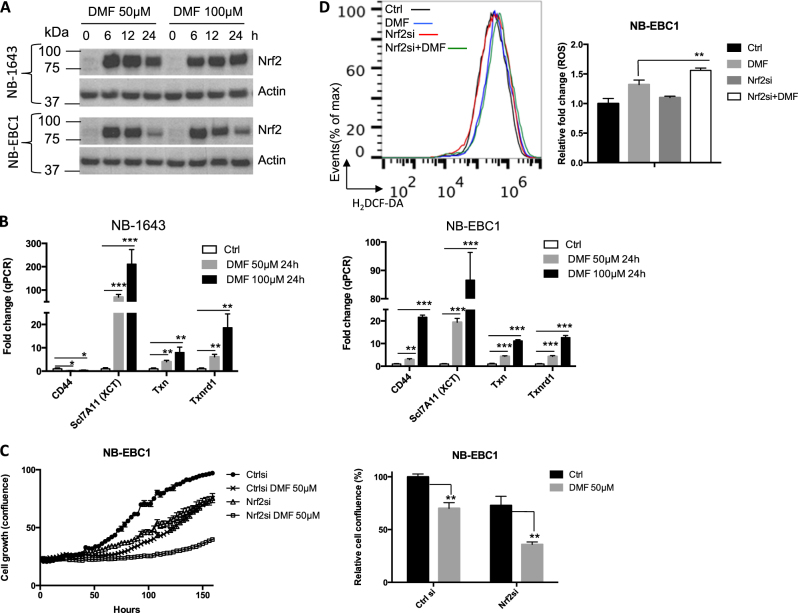
DMF induces a compensatory NRF2-mediated response. **a** The expression of indicated proteins in NB-1643 and NB-EBC1 cells following DMF treatment was determined by immunoblot. Data are representative of two independent experiments. **b** The mRNA levels of genes in Txn pathway were determined by qPCR. mRNA levels of genes in control cells were set to 1. Error bars represent standard deviation from the mean of triplicate samples. **c** NB-EBC1 cells were transfected with control or Nrf2 siRNA for 3 days and then treated with DMF. Cell proliferation was determined by live-cell imaging analysis (IncuCyte ZOOM™) (left panel). Cell confluences of NB-EBC1 cells transfected with control siRNA without DMF at 144 h were set to 100% (right panel). Error bars represent standard deviation from the mean of quadruplicate samples. **d** NB-EBC1 cells were transfected with control or Nrf2 siRNA for 2 days and then treated with 50 μM DMF for 1 h. The ROS level (left panel) was determined by flow cytometry in cells after 30 min incubation with 5 μM CM-H_2_DCFDA at 37 °C. The ROS levels in control siRNA-transfected cells were set to 1 in the bar graph (right panel). Error bars represent standard deviation from the mean of triplicate samples. Data are representative of two independent experiments

We have shown the induction of an enriched oxidative response gene signature in NBL cells following DMF treatment (Fig. S3B). Consistent with previous studies in other cells^23,24^, we found DMF treatment directly induced ROS levels in NBL cells (Fig. 4d). A central transcriptional mechanism of defending against oxidative stress is through the activation of nuclear factor erythroid 2-related factor 2 (NRF2), which drives the expression of a wide range of metabolic genes involved in producing, regenerating, and utilizing GSH. NRF2 is also involved in modulating thioredoxin (TXN), NADPH generation, and iron sequestration. The expression of these genes therefore converges on suppressing oxidative stress^28^. Previous studies suggested that DMF could covalently modify Kelch-like ECH-associated protein 1 and stabilize NRF2^29^. Consistent with this, DMF treatment significantly induced the expression of NRF2 at the protein level (Fig. 5a). In addition, the transcription of four canonical NRF2 targets was induced in NBL cells following DMF treatment in a dose-dependent and time-dependent manner (Fig. 5b). The siRNA-mediated knockdown of NRF2 partially suppressed the induction of three canonical NRF2 targets, CD44, Txnrd, and XCT upon DMF treatment (Fig. S5A). Nevertheless, the combination of NRF2 siRNA and DMF treatment led to a significantly higher level of ROS compared to DMF treatment alone (Fig. 5d). These results suggest that NRF2-mediated anti-oxidative response may protect cells from DMF treatment. Since DMF treatment suppresses NBL cell growth and MYCN expression in a ROS-dependent manner (Fig. 4e, f), we next assessed MYCN level and cell growth following the treatment of DMF, NRF2 siRNA or the combination of DMF and NRF2 siRNA. Our data showed that the combination of DMF and NRF2 siRNA resulted in the greatest suppression on MYCN levels (Fig S5B) and NBL cell growth (Fig. 5c). Taken together, our data suggest that DMF treatment readily induces a robust NRF2-mediated anti-oxidative response, which might play a compensatory role in defending against ROS induction following DMF treatment.

## Discussion

NBL like many other pediatric cancers arises from embryonal cell hyperplasia and therefore possesses embryonal features including the metabolic state associated with embryonic development^30^. A better understanding of the regulatory mechanism and the metabolic state of pediatric cancer is essential for identifying potential therapeutic targets that are unique for tumor but not for normal tissues in a developmental stage. Proto-oncogene Myc is required for metabolic reprogramming in most adult cancers and in immune cells^31–33^. The dysregulation of MYCN, a member of the Myc family of oncogenes that is primarily expressed in the early stage of embryonic development and required for neural crest development, is a driver of many high-risk NBL^30,34,35^. In the current study, we have revealed a tight association between MYCN overexpression and the transcriptional upregulation of metabolic genes in glutaminolysis. Interestingly, the partial knockdown of MYCN suppresses glutaminolysis, but does not affect glycolysis.

Conventional chemotherapies target biological processes involved in cell proliferation, including cell division and DNA replication, which can cause significant toxicity to normal tissues. Chemotherapeutics, particularly those that cause DNA damage, can also induce malignant transformation, leading to secondary cancers. Thus, novel therapeutic targets are being identified that are more specific to cancer cell biology. One such approach is to target cancer metabolism^4,31,36,37^. The metabolic processes are hallmarks of cancers^1,2^, and therapeutic inhibition of these processes inherently have fewer systemic toxicities^38^. Genomic amplification of the Myc oncogene family member, MYCN, occurs in about 50% of high-risk NBL and is the most prevalent genetic abnormality identified in NBL. MYCN is a potent oncogenic driver and the single worst prognostic biomarker in NBL, associated with <30% chance of survival^11^. Recent studies suggest that pharmacological targeting BRD4, a member of the BET bromodomain family, can indirectly suppress MYC expression in various cancer models. However, the direct pharmacological targeting MYCN, which functions as a transcription factor, remains a challenge^39^. As a key MYCN-regulated process, the metabolic program is a critical biological pathway in cancer and has been recently validated as a therapeutic target^38,40^. Previous work demonstrated that NBL tumors overexpressed ornithine decarboxylase (ODC), a key metabolic enzyme in polyamine biosynthetic pathway, and that proliferation of preclinical models of NBL were suppressed by ODC inhibitor α-difluoromethylornithine (DFMO)^41^. DFMO is currently used in two ongoing clinical trials to prevent or treat refractory and relapsed NBL, demonstrating the potential of targeting metabolic pathways^41–44^.

Cancer cells often display elevated levels of ROS compared with non-transformed cells. ROS are largely generated as by-products of the mitochondrial ETC, and is intimately involved in tumor initiation, tumor progression, and responses to therapy^21,22^. While low levels of ROS are involved in modulating oncogenic signaling pathways, promoting cancer tumor proliferation and survival, excessive ROS induces oxidative stress, which often renders tumors vulnerable to further stresses. Hence, strategic enhancement of oxidative stress either by augmenting ROS production or suppressing antioxidant capacity may kill cancer cells while sparing non-transformed cells^37,45–47^. We have shown that NBL cells are highly addicted to MYCN-driven glutaminolysis, which drives cell growth by providing bioenergetic support but also induces oxidative stress by producing ROS. Transient activation of MYCN enhances ROS production, indicating that MYCN-dependent glutaminolysis induces oxidative stress by producing ROS as a by-product. As such, we envision that the MYCN-dependent metabolic reprogramming and oxidative stress in NBL represents a critical metabolic vulnerability that could be exploited therapeutically by ROS augmenting agents. DMF treatment engaged a very rapid oxidative stress response in NBL cells, due to the augmentation of ROS production. ROS scavenger, NAC, annuls DMF-dependent suppression on cell growth. It has also been reported that DMF impacts the epigenetic machinery and nuclear factor-κB signaling in transformed cells^48,49^. DMF is a long-approved treatment for psoriasis in Germany^50,51^ and is approved by the FDA as a first-line oral treatment for relapsing MS (marketed as Tecfidera by Biogen)^52,53^. This long record of accomplishment in clinical use demonstrates that DMF is a safe drug with generally mild toxicities and may represent an optimal drug for future development.

## Materials and methods

### Reagents

Cell culture materials and Matrigel were purchased from Fisher Scientific Inc. (Waltham, MA, USA). Glucose-free and glutamine-free Dulbecco's modified Eagle's media, Dox, DMKG, DON, *N*-acetyl-l-cysteine, and GSH reduced ethyl ester were purchased from Sigma-Aldrich Inc. (Saint Louis, MO, USA). DMF was from ACROS Organics (Geel, Belgium). Chicken embryo extract was from Gemini Biological Products (West Sacramento, CA, USA). B-27 supplement and N-2 supplement were obtained from Gibco (Waltham, MA, USA). Anti-MYCN, anti-fumarate hydratase, anti-Nrf2, and anti-actin antibodies were from Santa Cruz Biotechnology (Santa Cruz, CA, USA).

### Cell culture

Cell line identities have been authenticated by short tandem repeat analysis. NB-1643 and NB-EBC1 cells were kindly provided by Dr. Peter Houghton. NB975A2, a mouse NBL cell line derived from spontaneous tumor of TH-MYCN mice, was a gift from Dr. Rimas Orentas. All NBL cells used were grown in Dulbecco’s modified Eagle’s medium with 10% fetal calf serum in a 37 °C humidified atmosphere of 95% air and 5% CO_2_. MycN3 cells (a gift of Jason Shohet, Houston, TX, USA) were grown in RPMI-1640 medium with 10% fetal calf serum in a 37 °C humidified atmosphere of 95% air and 5% CO_2_^20^. JoMa cells were grown on cell culture flask/dish coated with fibronectin, in NCC-medium supplemented with 10% chicken embryo extract, B-27 supplement, N-2 supplement, and 200 nm 4-OHT, and the medium was changed daily^18,19^.

### siRNA transfection

The siRNA oligonucleotides corresponding to human MYCN, FH, and Nrf2 were purchased from Fisher Scientific Inc. siRNA oligonucleotides (20 nM) were transfected into cells using Lipofectamine RNAiMAX reagent (Invitrogen). After 48–72 h of transfection, IBs were carried out to examine the knockdown of targeted proteins.

### Lentiviral transduction

Plasmids encoding MYCN shRNA or mouse MYCN were purchased from Transomic Technologies (Huntsville, AL, USA). Lentiviruses were produced with the helper vectors in HEK293FT cells kindly provided by Dr. Peter Hunghton according to the protocol^54^. Lentiviruses, encoding for inducible MYCN-shRNA expression or mouse MYCN expression, were directly added to the cell culture medium and incubated for 48–72 h before further analysis.

### RNA isolation, reverse transcription, and qPCR

Total RNA was isolated using the RNA extraction kit (Zymo and Qiagen) and was reverse transcribed using random hexamer and M-MLV Reverse Transcriptase (Invitrogen). SYBR green-based quantitative RT-PCR for specific genes was performed using the Applied Biosystems Real-Time PCR System. Samples for each experimental condition were run in triplicate and were normalized to β-2-microglobulin to determine relative expression levels. Primer sequences were obtained from PrimerBank^55^. Primer sequences are listed in Table S2.

### Protein extraction and western blot analysis

Cells were harvested, lysed, and sonicated at 4 °C in a lysis buffer (50 mM Tris-HCl, pH 7.4, 150 mM NaCl, 0.5% sodium dodecyl sulfate, 5 mM sodium pyrophosphate, protease and phosphatase inhibitor tablet). Cell lysates were centrifuged at 13,000 × *g* for 15 min, and the supernatant were recovered. The protein concentrations were determined by using the Pierce™ BCA Protein Assay kit (Thermo Fisher Scientific). After 5 min boiling in 4 × NuPAGE^®^ LDS Sample Buffer with 10× Reducing solution (Thermo Fisher Scientific), the proteins were separated by NuPAGE 4–12% Protein Gels (Thermo Fisher Scientific), transferred to PVDF membranes by using the iBlot Gel Transfer Device (Thermo Fisher Scientific), and probed with the appropriate primary antibodies. Membrane-bound primary antibodies were detected using secondary antibodies conjugated with horseradish peroxidase. IBs were developed on films using the enhanced chemiluminescence technique.

### Metabolic activity analysis

Glycolytic activity was determined by measuring the detritiation of [5-^3^H]glucose^56^. In brief, one million cells were suspended in 0.5 ml fresh media. The experiment was initiated by adding 1 μci [5-^3^H]glucose, and 2 h later, media were transferred to a 1.5 ml microcentrifuge tube containing 50 μl of 5 N HCl. The microcentrifuge tubes were then placed in 20 ml scintillation vials containing 0.5 ml water with the vials capped and sealed. ^3^H_2_O was separated from unmetabolized [5-^3^H]glucose by evaporation diffusion for 24 h at room temperature. A cell-free sample containing 1 μci [5-^3^H]glucose was included as a background control. Glutaminolysis was determined by the rate of ^14^CO_2_ released from [U-^14^C]glutamine^57^. In brief, one million cells were suspended in 0.5 ml fresh media. To facilitate the collection of ^14^CO_2_, cells were dispensed into 7 ml glass vials (TS-13028, Thermo) with a PCR tube containing 50 μl of 0.2 M KOH glued on the sidewall. After adding 0.5 μci [U-^14^C]glutamine, the vials were capped using a screw cap with rubber septum (TS-12713, Thermo). The assay was stopped 2 h later by injection of 100 μl of 5 N HCl and the vials were kept at room temperate overnight to trap the ^14^CO_2_. The 50 μl of KOH in the PCR tube was then transferred to scintillation vials containing 10 ml scintillation solution for counting. A cell-free sample containing 0.5 μci [U-^14^C]glutamine was included as a background control.

### Transcriptome analysis

NB-1643 cells were incubated with 100 μM DMF and harvested at designated time points for RNA extraction. RNA samples were then performed microarray by St. Jude Children’s Research Hospital. Microarray data were submitted with assigned GEO number as GSE98241.

### Chromatin immunoprecipitation-polymerase chain reaction

ChIP-PCR was done as previously prescribed^58^, using Magna EZ-ChIP (Millipore) according to the manufacturer’s protocol. Briefly, after 24-h induction of MYCN expression, the inducible MycN3 cells (a gift of Jason Shohet, Houston, TX, USA)^20^ were cross-linked with 3% paraformaldehyde for 10 min and quenched for 5 min with 125 mmol/l of glycine. After sonification, cell lysates were spun down and 100 µl of supernatant was diluted to 500 μl for immunoprecipitation with 5 μg of MYCN antibody (Santa Cruz). After serial washing, DNA–protein cross-links were reversed and DNA was extracted for PCR. The PCR primers were designed according to the MYCN ChIP-seq data (GSE94782) in MYCN-amplified NBL cells including NB-1643. Primer sequences are listed in Table S3.

### ROS assay

ROS levels were detected using the CM-H_2_DCFDA probe purchased from Thermo Fisher Scientific. Cells were incubated with 5 µM CM-H2DCFDA for 30 min in serum-free media at 37 °C and analyzed by flow cytometry immediately.

### Animal studies

C57BL/6NHsd mice were purchased from Envigo. All mice were kept in specific pathogen-free conditions within the research institute at Nationwide Children’s Hospital. Animal protocols were approved by the Institutional Animal Care and Use Committee of the research institute at Nationwide Children’s Hospital. NB975A2 cells mixed with 70% Matrigel were injected subcutaneously to mice for the establishment of NBL xenograft model. Mice were randomized into different groups when tumors were 100 to 200 mm^3^. For the control group, a vehicle control (0.8% hydroxyethyl cellulose) was given by oral gavage every day. For the treated group, DMF was administered orally at a dose of 300 mg/kg every day till the endpoint of 2,000 mm^3^. Tumor volumes were determined with the method of tumor length times tumor width times tumor width times 0.52.

### Ethics statement

All animal experiments were conducted in accordance with institutional animal care and use committee of the Research Institute at Nationwide Children’s Hospital approved protocols, designed to minimize the numbers of mice used and to minimize any pain or distress. The named institutional review board or ethics committee specifically approved this study.

### Statistical analysis

*P* values were calculated with Student’s *t* test. *P* values < 0.05 were considered significant, with *P*values <0.05, <0.01, and <0.001 indicated as *, **, and ***, respectively.

## Electronic supplementary material

Figure S1

Figure S2

Figure S3

Figure S4

Figure S5

Table S1

Table S2

Table S3
