# Supplementary material for: MYCN drives glutaminolysis in neuroblastoma and confers sensitivity to an ROS augmenting agent
Source: Cell Death Dis. 2018 Feb 14;9(2):220. doi: 10.1038/s41419-018-0295-5 (PMC5833827; doi:10.1038/s41419-018-0295-5)
Supplement: Supplementary file 1 — Figure S1 [file 41419_2018_295_MOESM1_ESM.pptx]

## Slide 1
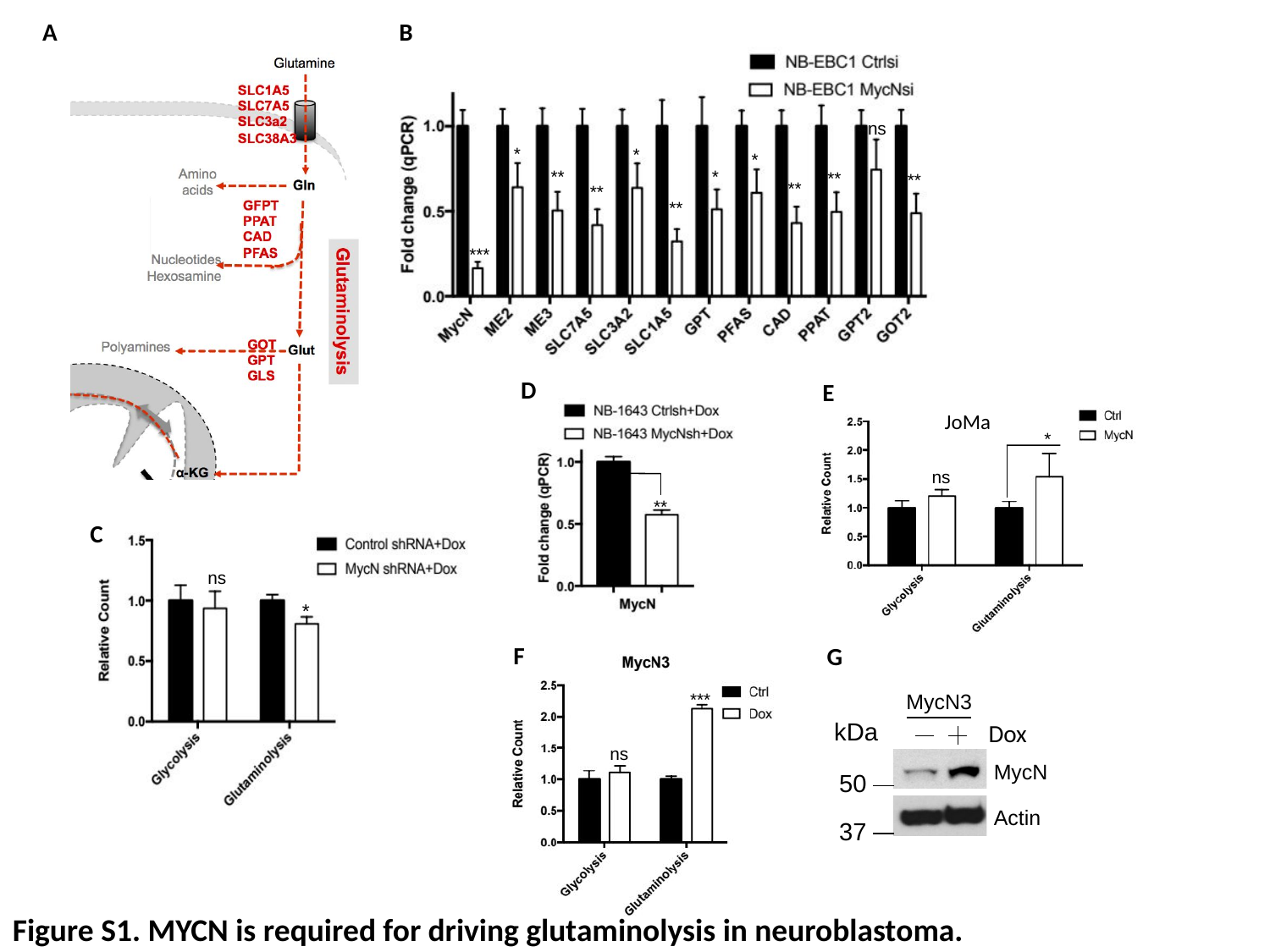

A
B
ns
*
*
*
**
*
**
**
**
**
**
***
D
**
E
*
ns
JoMa
C
ns
*
F
***
ns
G
MycN3
kDa
Dox
MycN
50
Actin
37
Figure S1. MYCN is required for driving glutaminolysis in neuroblastoma.
