# Supplementary material for: MYCN drives glutaminolysis in neuroblastoma and confers sensitivity to an ROS augmenting agent
Source: Cell Death Dis. 2018 Feb 14;9(2):220. doi: 10.1038/s41419-018-0295-5 (PMC5833827; doi:10.1038/s41419-018-0295-5)
Supplement: Supplementary file 2 — Figure S2 [file 41419_2018_295_MOESM2_ESM.pptx]

## Slide 1
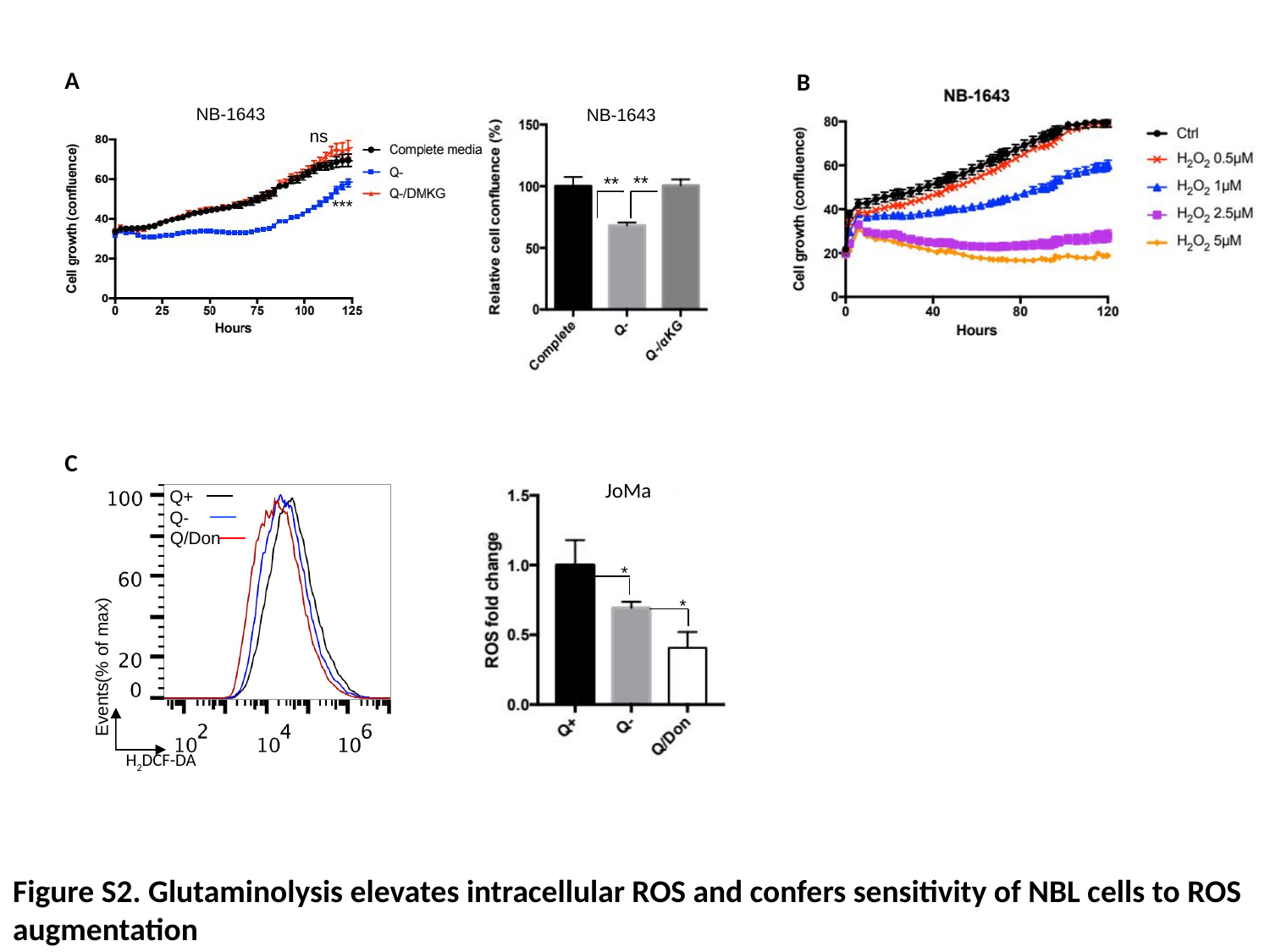

A
**
**
ns
***
NB-1643
NB-1643
B
C
*
*
Q+
Q-
Q/Don
Events(% of max)
H2DCF-DA
JoMa
Figure S2. Glutaminolysis elevates intracellular ROS and confers sensitivity of NBL cells to ROS augmentation
