# Supplementary material for: MYCN drives glutaminolysis in neuroblastoma and confers sensitivity to an ROS augmenting agent
Source: Cell Death Dis. 2018 Feb 14;9(2):220. doi: 10.1038/s41419-018-0295-5 (PMC5833827; doi:10.1038/s41419-018-0295-5)
Supplement: Supplementary file 3 — Figure S3 [file 41419_2018_295_MOESM3_ESM.pptx]

## Slide 1
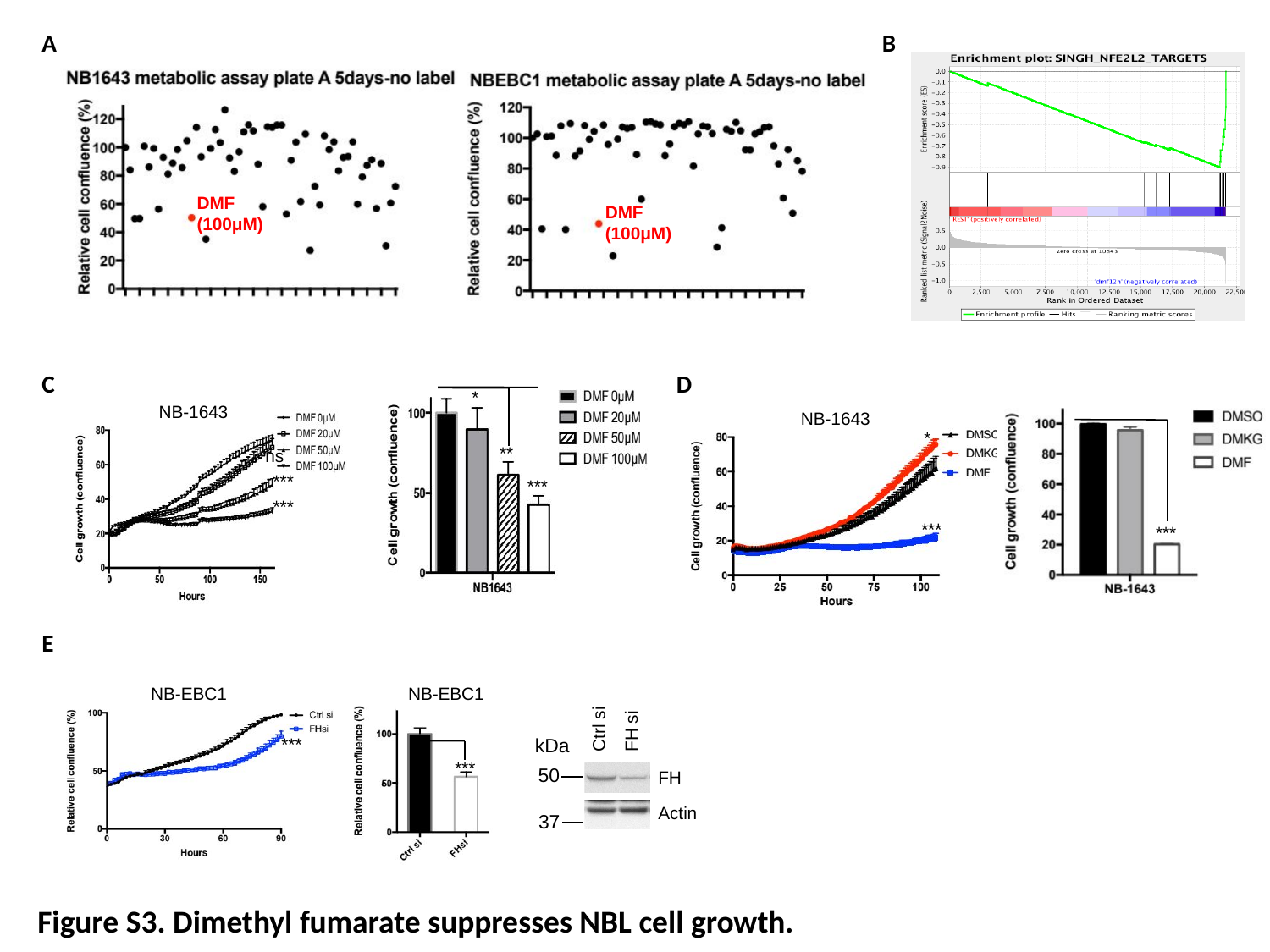

A
B
DMF
(100μM)
DMF
(100μM)
C
*
**
ns
***
***
***
NB-1643
D
*
***
***
NB-1643
E
Ctrl si
FH si
kDa
50
FH
Actin
37
***
***
NB-EBC1
NB-EBC1
Figure S3. Dimethyl fumarate suppresses NBL cell growth.
