# Supplementary material for: MYCN drives glutaminolysis in neuroblastoma and confers sensitivity to an ROS augmenting agent
Source: Cell Death Dis. 2018 Feb 14;9(2):220. doi: 10.1038/s41419-018-0295-5 (PMC5833827; doi:10.1038/s41419-018-0295-5)
Supplement: Supplementary file 4 — Figure S4 [file 41419_2018_295_MOESM4_ESM.pptx]

## Slide 1
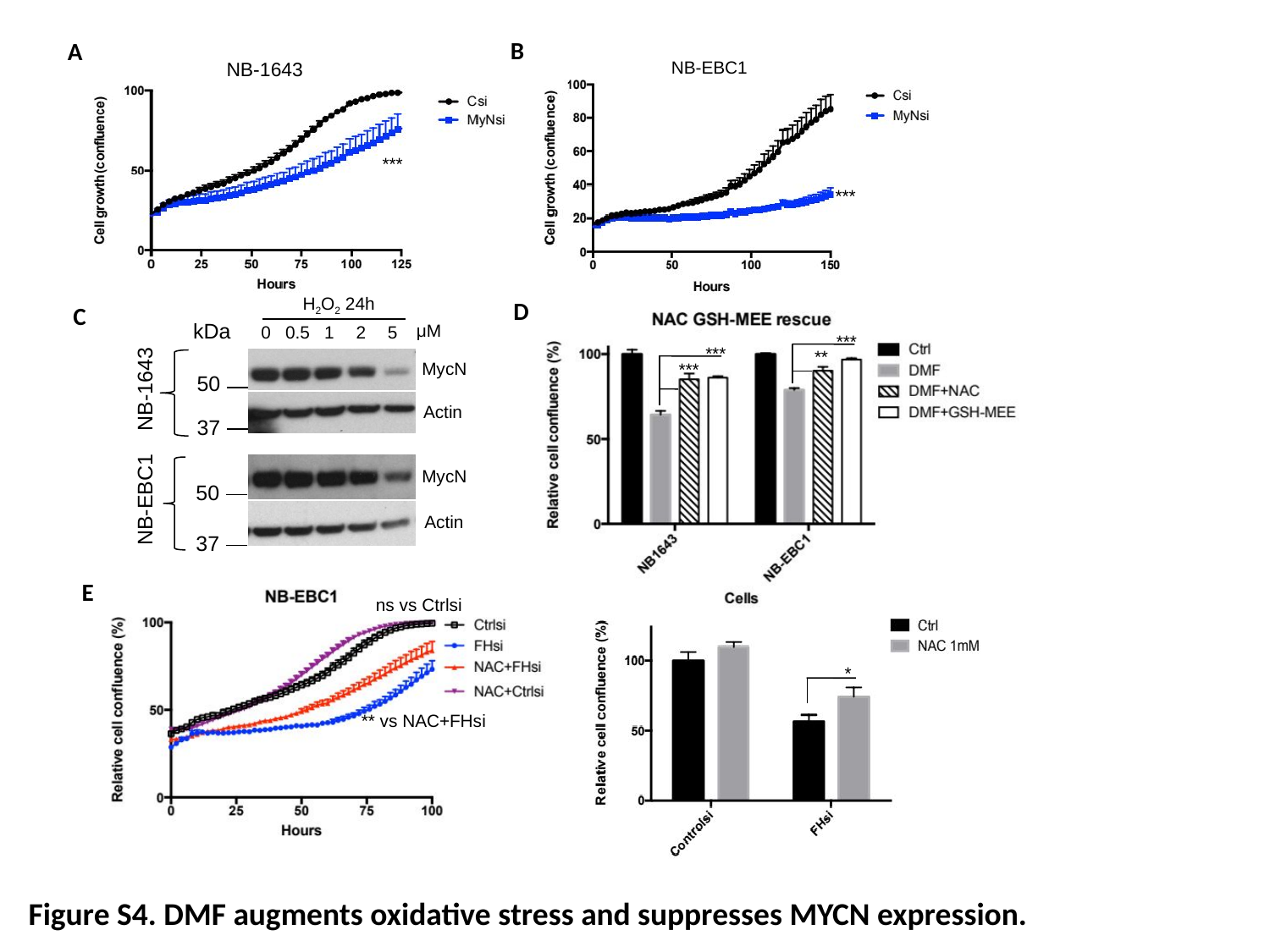

B
***
NB-EBC1
A
NB-1643
***
H2O2 24h
C
kDa
μM
0
0.5
1
2
5
MycN
50
NB-1643
Actin
37
MycN
50
NB-EBC1
Actin
37
D
***
***
 **
***
E
ns vs Ctrlsi
 *
** vs NAC+FHsi
Figure S4. DMF augments oxidative stress and suppresses MYCN expression.
