# Supplementary material for: MYCN drives glutaminolysis in neuroblastoma and confers sensitivity to an ROS augmenting agent
Source: Cell Death Dis. 2018 Feb 14;9(2):220. doi: 10.1038/s41419-018-0295-5 (PMC5833827; doi:10.1038/s41419-018-0295-5)
Supplement: Supplementary file 5 — Figure S5 [file 41419_2018_295_MOESM5_ESM.pptx]

## Slide 1
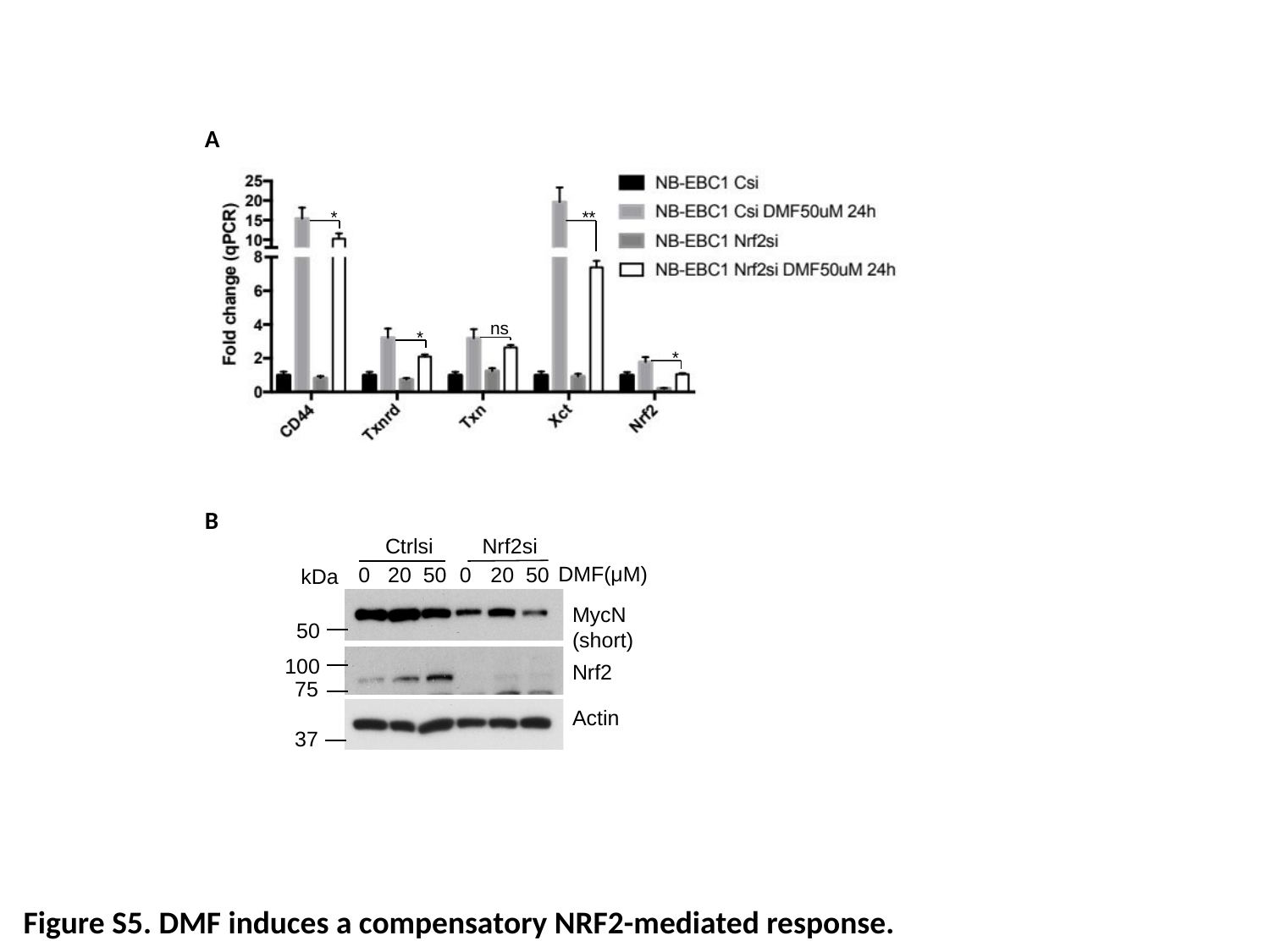

A
 *
 **
 ns
 *
 *
B
Ctrlsi
Nrf2si
DMF(μM)
0
20
50
0
20
50
kDa
MycN (short)
50
100
Nrf2
75
Actin
37
Figure S5. DMF induces a compensatory NRF2-mediated response.
